# Hospital Festivals and Meetings

**Published:** 1894-07-07

**Authors:** 


### HOSPITAL FESTIVALS AND MEETINGS.

### MILLER HOSPITAL AND ROYAL KENT DISPENSARY, GREENWICH.

The annual general meeting of the governors of this hospital was held on June 27th, with Mr. E. Sydney in the chair. The work of the institution has gone on increasing, the number of patients attended during the past nine months having reached a total of 11,118, of which 203 were in-patients. Unfortunately, the income of the charity has not expanded in the same degree, and one of the resolutions passed at the meeting empowered the trustees to sell £2,000 for the purpose of clearing off the debts of the institution. The Chairman, in moving the adoption of the report, said that extra expense had been incurred during the past year by the necessity of acquiring the stables adjoining the hospital, a step which was unavoidable for sanitary considerations. On the space thus acquired it was the hope and intention of the committee to erect not only a room which would enable the pressure on the existing out-patient and casualty department to be somewhat relieved, but also, if possible, two additional wards. In answer to a question, he further stated that the committee had no thought of commencing these operations before the necessary funds had been obtained; but he wished to make the need of the proposed extension apparent to the public.

An alteration in the rules rendering ineligible for service on the committee more than eleven members of the medical staff was objected to by Mr. Thomas Moore, who proposed an amendment, subsequently carried, excepting the honorary consulting physicians and surgeons from the application of the rule.

Votes of thanks to the honorary secretary, Major-General Roberts, and the hon. medical staff were passed, and the Chairman expressed the gratitude of the committee to those ladies and gentlemen who had been active during the past year in organising dramatic performances for the benefit of the institution. Among those present were Mr. F. G. Cox, Mr. A. C. Haycraft, Mr. Fox Batley, Mr. Taylor, and Major-General Roberts.

